# Prevalence, Morphometric Characteristics of the Accessory Abductor Pollicis Longus Muscle and Clinical Implications: A Cadaveric Study

**DOI:** 10.3390/diagnostics15192455

**Published:** 2025-09-25

**Authors:** Jhonatan Duque-Colorado, Victor Hugo Rodriguez-Torrez, Laura García-Orozco, Rubén Daniel Algieri, Nicolás E. Ottone

**Affiliations:** 1 Doctoral Program in Morphological Sciences, Faculty of Medicine, Universidad de La Frontera, Temuco 4780000, Chile; jhonatanandresduquecolorado@gmail.com (J.D.-C.); l.garcia05@ufromail.cl (L.G.-O.); 2 Center of Excellence in Morphological and Surgical Studies, Universidad de La Frontera, Temuco 4811230, Chile; 3 Medicine and Dentistry Department, Universidad Privada del Valle, La Paz 5911, Bolivia; 4 III Chair of Anatomy, Department of Anatomy, Faculty of Medicine, Universidad de Buenos Aires, Buenos Aires C1121ABG, Argentina; rdalgieri08@hotmail.com; 5 Laboratory of Plastination and Anatomical Techniques, Universidad de La Frontera, Temuco 4811230, Chile; 6 Adults Integral Dentistry Department, Center for Research in Dental Sciences (CICO), Faculty of Dentistry, Universidad de La Frontera, Temuco 4811230, Chile

**Keywords:** morphometry, anatomy, anatomical variation, forearm, muscle, abductor pollicis longus

## Abstract

**Background/Objectives:** The abductor pollicis longus (APL) muscle exhibits a high degree of anatomical variation, particularly in the number and configuration of its tendons. Understanding these variants is crucial in surgical contexts, especially for tendon transfer and reconstruction procedures. This study aimed to determine the prevalence and morphometric characteristics of the accessory abductor pollicis longus (AAPL) muscle in a Bolivian cadaveric population. **Methods:** A descriptive, cross-sectional study was performed on 16 forearms from eight adult cadavers (six males and two females) preserved in 10% formalin. Cadaveric dissection was conducted following the AQUA guidelines, with measurements obtained for the AAPL proximal tendon length (PTL), distal tendon length (DTL), muscle length (ML), and transverse muscle length (TML) using a digital caliper. Statistical analysis was carried out using SPSS v26. **Results:** The AAPL muscle was present in 50% of forearms. Most were unilateral, with one bilateral case. The muscle exhibited a fusiform shape, with fibers aligned longitudinally. Morphometric analysis revealed a mean PTL of 1.20 ± 0.08 cm, DTL of 3.91 ± 0.52 cm, ML of 5.30 ± 0.45 cm, and TML of 0.55 ± 0.056 cm. One case (6.25%) exhibited a multicaudal APL with an additional tendon measuring 6.23 cm. No significant correlations were found between muscle and tendon measurements. **Conclusions:** AAPL muscles are relatively common and demonstrate notable morphometric variation. While the proximal tendon may be inadequate for grafting due to its short length, the distal tendon offers a viable alternative for reconstructive procedures. Recognition of such variants is clinically relevant, as they may contribute to pathologies like De Quervain’s tenosynovitis or serve as graft sources in surgical interventions.

## 1. Introduction

The abductor pollicis longus (APL) is part of the deep layer of the extensor compartment of the forearm, originating distal to the anconeus, on the posterior surface of the ulna diaphysis, in the interosseous membrane, and the middle third of the posterior surface of the radius. The muscle then descends, becoming superficial in the distal third of the forearm, to insert into the lateral face of the first metacarpal and the trapezium bone [1].

Commonly, APL is used for forming tendinous interconnections in surgical procedures, such as resection-suspension arthroplasty in rhizarthrosis [2,3,4]. Although the flexor carpi radialis is also involved in this surgical procedure, the APL is preferred, since the kinematics of the carpus are less altered with it [5,6], thanks to the variability it presents in its number of tendons. However, studies have shown that the use of APL in this type of procedure generates a reduction in several parameters associated with its functionality [7].

In this context, understanding the anatomical variants of this muscle is essential, as they may provide a valuable source of graft material, particularly for suspension arthroplasty following trapeziectomy in rhizarthrosis and for tendon reconstruction. Thus, the objective of this study was to identify the prevalence and morphometric characteristics of accessory abductor pollicis longus (AAPL) in cadaveric samples.

## 2. Materials and Methods

A descriptive, quantitative, non-experimental, cross-sectional study was conducted, based on the analysis of cadaveric specimens. The methodological planning of this research followed the Anatomical Quality Assurance (AQUA) checklist: guidelines for reporting original anatomical studies [8].

We dissected a total of 16 forearms (eight left and eight right), corresponding to eight adult cadavers (six males and two females) from the cadaveric donation program of Universidad del Valle, La Paz Campus. This donation program complies with both the Declaration of Helsinki and national legal and ethical requirements. We performed the dissection procedure between April 2024 and February 2025. The cadavers had been previously preserved in a 10% formaldehyde solution. We included only forearms that showed no signs of injury or previous surgeries that could affect the area of interest in the analysis.

### 2.1. Cadaveric Dissection

We dissected the posterior compartment of the forearm and wrist, as well as the dorsum of the hand, in the cadaveric specimens. First, we made a skin incision and reflected the skin along with the subcutaneous tissue and superficial fascia to expose the muscles of the superficial layer. These structures were carefully separated to access the deep layer, where we examined the AAPL, including its presence, shape, origin, and insertion.

### 2.2. Morphometric Analysis

**Figure 1:**
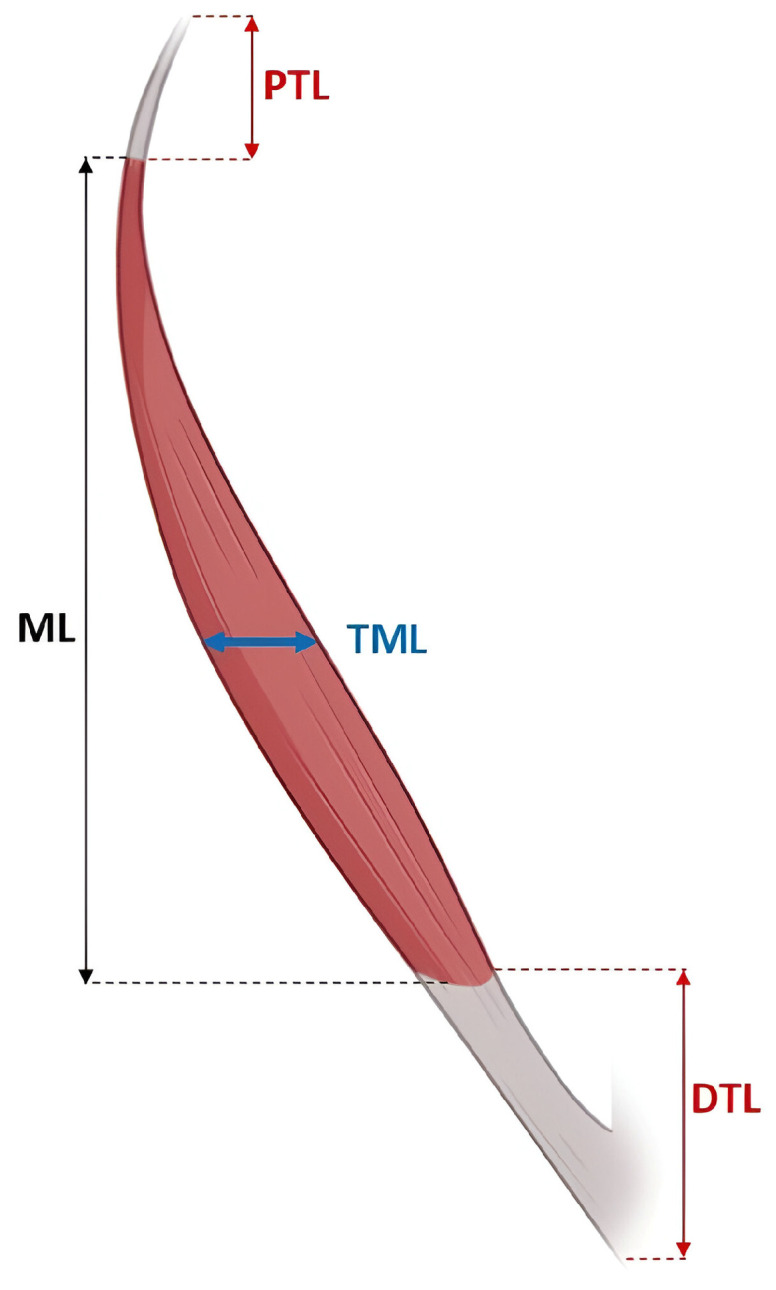
Different morphometric measurements made to the accessory abductor pollicis longus, proximal tendon length (PTL), muscle length (ML), transverse muscle length (TML), and distal tendon length (DTL).

We performed the following morphometric measurements on each AAPL: (1) Proximal tendon length (PTL), defined as the distance from the point of tendon origin to the proximal myotendinous junction; (2) Distal tendon length (DTL), defined as the distance from the distal myotendinous junction to the final tendon insertion point; (3) Muscle length (ML): distance between the proximal and distal myotendinous junctions, following the longitudinal course of the muscle belly; (4) Transverse muscle length (TML): maximum length of the muscle belly, measured perpendicular to the longitudinal axis, at its midpoint (Figure 1). Each measurement was performed three times by the same researcher, using a TOTAL TMT321501 digital caliper (TOTAL TOOLS CO., PTE. LTD., Stgo, Chile ) with an accuracy of 0.01 mm.

### 2.3. Statistical Analysis

The results were recorded in a database generated in Microsoft Excel 2019 software (Microsoft, Redmond, WA, USA) and analyzed in the SPSS statistical software, version 26 (IBM Corp., Armonk, NY, USA). Measurements were expressed as the mean (standard deviation, SD) or median, depending on the data distribution. Categorical variables appear as absolute values (n) and percentages (%).

The normality of the data obtained for each variable was evaluated using the Kolmogorov–Smirnov test. Subsequently, the differences between PTL and DTL were analyzed using Student’s t-test or the Mann–Whitney U test. Pearson’s or Spearman’s correlation tests were applied, depending on the data distribution, to evaluate the associations of ML and TML with the other morphometric parameters (PTL and DTL), with a significance level of α = 0.05.

## 3. Results

The AAPL muscle was present in 50% (n = 8) of the forearms analyzed (six males and two females), of which six cases were unilateral (two left and four right) and one bilateral. In all cases, the AAPL exhibited the characteristics of a fusiform muscle, with a wider muscle belly that narrowed toward its ends. Furthermore, its muscle fibers ran parallel to the muscle’s longitudinal axis.

**Figure 2:**
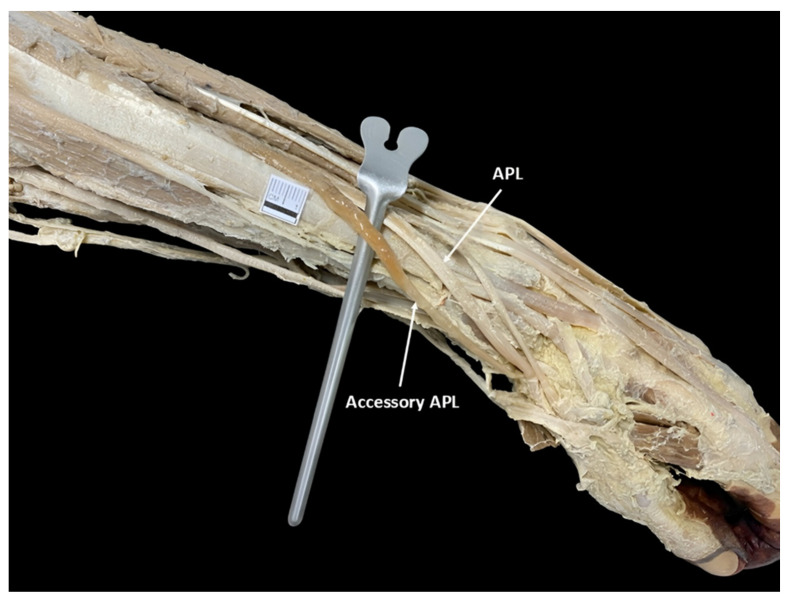
The accessory abductor pollicis longus (APL) as an independent structure, adjacent to the APL muscle.

**Figure 3:**
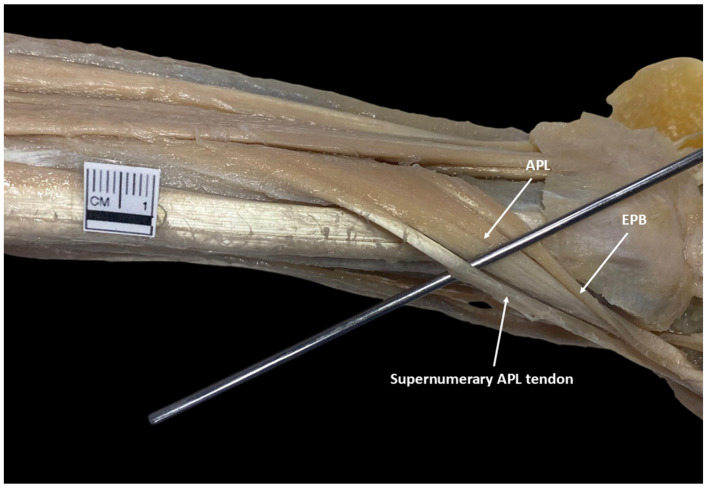
Multicaudal abductor pollicis longus (APL), adjacent to the extensor pollicis brevis (EPB), with a supernumerary tendon emerging from its lateral margin.

In the observed cases, the AAPL originated as an independent structure on the posterior surface of the radius and ulna, at the level of the middle third of the forearm (Figure 2). Its point of origin was situated above the origin site of the APL, characterized by its consistent and distinct arrangement relative to the nearby muscular structures. Regarding its insertion, it was situated at the base of the first metacarpal, lateral to the APL tendon. On the other hand, a multicaudal APL was identified in a single case (6.25%), corresponding to the left forearm of a male individual (Figure 3). The supernumerary tendon originated from the lateral margin of the muscle belly and inserted adjacent to the principal tendon, at the base of the first metacarpal.

Regarding morphometric parameters, significant differences were found between PTL and DTL (*p* < 0.001), with DTL being greater. Similarly, the ML, with a mean of 5.30 ± 0.45, was superior to the TML, whose mean was 0.55 ± 0.056, supporting the classification of the muscle as long. Regarding correlations, the analysis showed no significant association between ML and the evaluated morphometric parameters, including PTL, DTL, and TML (*p* > 0.05). Likewise, no correlation emerged between TML and the PTL and DTL parameters (*p* > 0.05). All measurement data appear in Table 1. Regarding the multicaudal APL, the only measurement recorded was the length of the supernumerary tendon, which had a DTL of 6.23 cm.

## 4. Discussion

Several studies have reported variations in the APL and its prevalence (Table 2), which include descriptions of a digastric APL [9], biceps [10], and multicaudal [10,11]. However, reports on the AAPL muscle remain scarce in the literature. Most available descriptions are limited to case reports involving individuals of Indian [12] and British [13] origin, in which the AAPL has been described as having a distinct origin, muscle belly, and insertion, independent of the principal APL muscle. These observations are consistent with our findings, as the AAPL originated superior to the origin of the main APL and was inserted lateral to its tendon. The anatomical and prevalence differences among these muscle variations are likely due to developmental alterations occurring during the embryonic stage. Although the formation of the upper limbs is regulated by the expression of Pax3, MyoD, Myf5, and MET [14], it has been shown that mutations in TBX5, specifically in the muscular connective tissue, cause modifications in the cleavage of the muscle bundles [15]. This process directly affects the APL, since at Carnegie stage 19 (CS19), the APL and the extensor pollicis brevis form a single muscle bundle located in the dorsal zeugopod of the upper limb. According to Wilde et al. [16], the division of this bundle begins at stage CS20, following a distal to proximal direction and with an ulnar orientation, until forming two differentiated muscle bundles at CS23. Consequently, alterations in TBX5 could lead to different muscle bundle segmentation patterns, such as the absence of segmentation resulting in a digastric APL, a partial segmentation resulting in a biceps APL, or as observed in our study, a muscle branching forming a multicaudal APL, as well as a supplementary segmentation forming an AAPL. Furthermore, Deshmukh et al. [17] suggested that these variations may also reflect persistent embryological insertion patterns, specifically toward the metacarpal, trapezium, and thenar muscles, regulated by molecular signals such as BMP4, WNT, and SHH.

Anatomical variants of APL, such as the biceps and multicaudal morphotypes, characterized by the presence of multiple tendons, have been considered as risk factors for the development of trapeziometacarpal osteoarthritis [19], intersection syndrome [20], and De Quervain’s tenosynovitis [12]. In this context, we propose that the presence of an AAPL may contribute to the development of these pathologies. The additional distal tendon could increase the structural complexity of the first extensor compartment, disrupt normal biomechanics, promote friction between tendon structures, and lead to functional overload. Recent anatomical studies have confirmed that the APL muscle exhibits a high degree of morphological variability, particularly in the number and distribution of its tendons. Marí-Gorreto et al. [21] found that 96.67% of upper limb specimens presented multiple tendinous slips, most commonly inserting into the abductor pollicis brevis, trapezium, or opponens pollicis muscles. Similarly, Sheth et al. [22] reported that 90% of dissected APLs had multiple tendons, ranging from two to seven, with the base of the first metacarpal as the most consistent insertion site, followed by slips to thenar muscles and fascia. Deshmukh et al. [17] supported these findings by identifying between one and five tendons per APL, with 93% inserting into the first metacarpal base and 7% into the trapezium. These considerations underscore the importance of detailed knowledge of such variations during clinical assessment, particularly in the affected populations.

The distal APL tendon, according to Rosas et al. [23], in the absence of the palmaris longus muscle, is used for tendon reconstruction. In addition, other surgical procedures in the hand are considered, such as suspension arthroplasty by resection in rhizarthrosis [2,3,4]. However, its use compromises several functional parameters, reducing the range of motion of the thumb, thumb opposition, tip pinch strength, palmar pinch strength, and lateral pinch strength [7]. In this context, although the presence of the AAPL could constitute a risk factor for the development of various pathologies, it could also be considered an anatomical variant of clinical utility, since being an accessory muscle that fulfills the same function as the APL, the use of its tendons for tendon reconstruction or other surgical procedures would not affect the kinematics of the thumb to a greater extent. Additionally, its effects could be further reduced when the tendon bands used are from the medial portion of the tendon, thus minimizing the impact on thumb abduction and stability.

While tendon position is a relevant factor in planning surgical interventions, surgeons must also evaluate other parameters, such as tendon length and muscle belly dimensions. The required minimum length for reconstructive procedures varies according to the tendon selected and the specific demands of the surgical technique. However, a minimum length of 1.5 cm is generally sufficient to achieve adequate graft consolidation and fixation [24]. Thus, the proximal tendon of the AAPL would not be considered a viable option for reconstructive procedures, given that its length of 1.40 cm, with a mean of 1.20 ± 0.080 cm, does not reach the reference values. In contrast, the distal tendon, with a maximum length of 6.95 cm and a mean of 3.91 ± 0.52 cm, clearly exceeds this threshold, making it the most suitable option compared to the proximal tendon. Others morphometric analyses revealed that tendons inserting on the first metacarpal tend to be longer, whereas medial slips or those originating from supernumerary muscles are significantly narrower [21]. The AAPL tendons described by Deshmukh et al. [17] had a comparable length but reduced thickness and width, which may limit their mechanical contribution but enhance their utility as graft material. Bilateral asymmetry in the number and dimensions of tendons was noted by both Sheth et al. [22] and Deshmukh et al. [17], highlighting the importance of individualized anatomical assessment. In surgical contexts, accessory tendons have proven valuable in reconstructive procedures, including trapeziometacarpal arthroplasty, given their favorable orientation and sufficient length [21,22]. In surgical contexts, accessory tendons have proven valuable in reconstructive procedures, such as trapeziometacarpal arthroplasty, due to their favorable orientation and sufficient length [21,22]. They have also been applied as grafts in the reconstruction of chronic extensor pollicis longus ruptures, with the morphometric characteristics of the distal AAPL tendon supporting its suitability as an optimal option. At two-year follow-up, patients undergoing this procedure showed significant functional improvement and high satisfaction [25], supporting its potential as a viable long-term anatomical alternative. Furthermore, high-resolution ultrasound can detect these anatomical variants with great accuracy, supporting their incorporation into preoperative planning. However, we did not find studies providing evidence on other properties, such as tensile strength. Therefore, further research is needed to determine the maximum load, deformation, and elastic modulus of this tendon.

Regarding the dimensions of the muscle belly, Gulati et al. [26] reported that the APL has an ML of 15.37 cm, a value higher than that observed in the present study for the AAPL. In the case of the TML, no previous references were found that would allow contrasting the obtained mean of 0.55 ± 0.056 cm. These findings support the proposal that surgical procedures should be performed preferentially on the tendons of the AAPL, given that, as a smaller muscle, its participation in abduction, extension, and stabilization movements of the thumb would be limited compared to the principal APL.

Understanding the relationships among the morphometric characteristics of the AAPL is essential for evaluating its functional viability in surgical procedures, such as tendon transfers, and for predicting its biomechanical impact on thumb movements. Since no significant correlations were found between the variables PTL, DTL, ML and TML, the evaluation of these characteristics should be performed independently, either using computed tomography or more accessible tools, such as ultrasound, which provide valuable information for clinical diagnosis, thus contributing to risk reduction and more effective surgical planning [27,28].

## 5. Conclusions

The AAPL muscle was present in 50% of the analyzed forearms, exhibiting notable morphometric variability and diverse anatomical configurations, including one case of a multicaudal variant. These findings reinforce the concept that APL morphological variations are common and may originate from developmental alterations during embryogenesis. Specifically, variations such as the presence of an accessory or multicaudal APL could be explained by differential expression or mutation of regulatory genes like TBX5 or persistent embryological insertion patterns mediated by BMP4, WNT, and SHH signaling pathways.

Clinically, these anatomical variants are highly relevant. The presence of additional tendons may increase compartmental complexity, contributing to conditions such as De Quervain’s tenosynovitis. At the same time, accessory tendons may serve as valuable graft material in reconstructive surgeries, particularly when the palmaris longus is absent. However, due to the short PTL, it is not suitable for grafting purposes. In contrast, the distal tendon of the AAPL exceeds the minimal length required for grafting and may offer a viable alternative without significantly compromising thumb function.

These results highlight the importance of thorough anatomical assessments, ideally using imaging tools such as ultrasonography, in both diagnostic and preoperative planning. Understanding the frequency, morphology, and clinical implications of APL variations enhances surgical outcomes and reduces the risk of iatrogenic complications. Future studies with larger and more diverse samples will be essential to confirm the prevalence and morphometric variability observed in our population.

## Figures and Tables

**Table 1:** Morphometric parameters of the accessory abductor pollicis longus.

Parameter	Minimum–Maximum (cm)	Mean ± SD (cm)
PTL	0.68–1.41	1.20 ± 0.080
DTL	2.33–6.95	3.91 ± 0.52
ML	2.78–7.28	5.3 ± 0.45
TML	0.33–0.78	0.55 ± 0.056

PTL: proximal tendon length; DTL: distal tendon length; ML: muscle length; TML: transverse muscle length; SD: standard deviation.

**Table 2:** Frequency and types of APL variants.

Authors	Population	Morphological Classification of APL	Prevalence (%)	Specimens Analyzed (%)
Fabrizio & Clemente [9]	North American	Digastric	30	50
Karauda et al. [10]	Polish	Biceps Multicaudal	24 50	50
Prameela et al. [11]	Indian	Multicaudal	80.90	115
Perruisseau-Carrier et al. [18]	French	Biceps	73	11
This study	Bolivian	Accessory muscle Multicaudal	43.75 6.25	16

APL: abductor pollicis longus.

## Data Availability

The data presented in this study are available on request from the corresponding author.

## Abbreviations

The following abbreviations are used in this manuscript:

APL	Abductor pollicis longus
AAPL	Accessory abductor pollicis longus
DTL	Distal tendon length
ML	Muscle length
TML	Transverse muscle length
EBP	Extensor pollicis brevis

## References

[1] Standring S. (2015). Gray’s Anatomy: The Anatomical Basis of Clinical Practice.

[2] Chang E.Y., Chung K.C. (2008). Outcomes of trapeziectomy with a modified abductor pollicis longus suspension arthroplasty for the treatment of thumb carpometacarpal joint osteoarthritis. Plast. Reconstr. Surg..

[3] Lee H.J., Kim P.T., Deslivia M.F., Jeon I.H., Lee S.J., Nam S.J. (2015). Results of abductor pollicis longus suspension ligamentoplasty for treatment of advanced first carpometacarpal arthritis. Clin. Orthop. Surg..

[4] Satria O., Wibowo R.S., Putra G.U., Fathurrahman I. (2023). Suture suspension sling arthroplasty in thumb carpometacarpal joint arthritis: A case series. Int. J. Surg. Case Rep..

[5] Rab M., Gohritz A., Gohla T., Krimmer H., Lanz U. (2006). Ergebnisse nach Resektions-Suspensions-Arthroplastik bei Rhizarthrose: Vergleich der Abductor pollicis longus-mit der Flexor carpi radialis-Sehnensuspension [Long-term results after resection arthroplasty in patients with arthrosis of the thumb carpometacarpal joint: Comparison of abductor pollicis longus and flexor carpi radialis tendon suspension]. Handchir. Mikrochir. Plast. Chir..

[6] Bravo E., Barco R., Bullón A. (2010). Anatomic study of the abductor pollicis longus: A source for grafting material of the hand. Clin. Orthop. Relat. Res..

[7] Hatipoğlu M.Y., Yapar A., Ergişi Y., Tokgöz M.A., Yapar D., Öztürk A.M. (2022). What is the clinical and functional effect of performing suspension arthroplasty with abductor pollicis longus tendon slip to carpometacarpal joint osteoarthritis of the thumb?. Jt. Dis. Relat. Surg..

[8] Tomaszewski K.A., Henry B.M., Kumar Ramakrishnan P., Roy J., Vikse J., Loukas M., Tubbs R.S., Walocha J.A. (2017). Development of the Anatomical Quality Assurance (AQUA) checklist: Guidelines for reporting original anatomical studies. Clin. Anat..

[9] Fabrizio P.A., Clemente F.R. (1996). A Variation in the Organization of Abductor Pollicis Longus. Clin. Anat..

[10] Karauda P., Olewnik Ł., Podgórski M., Polguj M., Ruzik K., Szewczyk B., Topol M. (2020). Anatomical Variations of the Abductor Pollicis Longus: A Pilot Study. Folia Morphol..

[11] Prameela M.D., Prabhu L.V., Murlimanju B.V., Pai M.M., Rai R., Kumar C.G. (2022). Morphological Variants of the Abductor Pollicis Longus and Extensor Pollicis Brevis Tendons: A Cadaveric Study. Muscles Ligaments Tendons J..

[12] Kaur A. (2024). Accessory Abductor Pollicis Longus: An Anatomical Case Report. J. Anat. Soc. India.

[13] Jaber N.K., Charrier C., Maouloud N., Legendre O., Sam F. (2025). Multiple Musculotendinous Variations in the Limbs: A Cadaveric Case Report. Cureus.

[14] García-Orozco L., Duque-Colorado J., Alarcón-Apablaza J., Roa I., Rojas M. (2024). Striated Musculature: Embryonic and Fetal Development. Int. J. Morphol..

[15] Besse L., Sheeba C.J., Holt M., Labuhn M., Wilde S., Feneck E., Bell D., Kucharska A., Logan M.P.O. (2020). Individual Limb Muscle Bundles Are Formed through Progressive Steps Orchestrated by Adjacent Connective Tissue Cells during Primary Myogenesis. Cell Rep..

[16] Wilde S., Feneck E.M., Mohun T.J., Logan M.P.O. (2021). 4D Formation of Human Embryonic Forelimb Musculature. Development.

[17] Deshmukh V., Talhar S., Muthiyan G., Kasat P., Chandrasekaran K., Sontakke B. (2023). Insights with consensus on Abductor Pollicis Longus from the Central Indian population at Nagpur, Maharashtra. Bioinformation.

[18] Perruisseau-Carrier A., Artz M., Ta P., Seizeur R., Hu W., Le Nen D. (2023). Feasibility of the Abductor Pollicis Longus Hemitendon Transfer for Thumb Opposition: An Anatomical Study. Orthop. Traumatol. Surg. Res..

[19] Opreanu R.C., Wechter J., Tabbaa H., Kepros J.P., Baulch M., Xie Y., Lackey W., Katranji A. (2010). Anatomic Variations of the First Extensor Compartment and Abductor Pollicis Longus Tendon in Trapeziometacarpal Arthritis. Hand.

[20] White A.C., Byrd J.J., McCumber T.L., Snow E.L. (2023). Prevalence, Evaluation, and Clinical Implications of a Reticular Tunnel Formed by Uncharacteristic Distal Fibers of the Abductor Pollicis Longus. Transl. Res. Anat..

[21] Marí-Gorreto J., San-Millán M., Carrera A., Tubbs R.S., Iwanaga J., Cateura A., Acquabona L., Reina M.A., Reina F. (2023). The anatomy of the tendon of abductor pollicis longus and its morphological variations: An anatomical approach emphasizing the clinical relevance. Ann. Anat..

[22] Sheth A.B.N., Patra A., Mahajan D. (2024). Anatomical variations and developmental insights of tendons in the first extensor compartment of the hand: Cadaveric study with surgical implications. Clin. Ter..

[23] Rosas S., Mesa C., Mesa F. (2017). The Abductor Pollicis Longus Tendon as an Alternative Graft in Hand Surgery. J. Hand Surg. Am..

[24] Guglielmetti L.G.B., Shimba L.G., do Santos L.C., Severino F.R., Severino N.R., de Moraes Barros Fucs P.M., de Paula Leite Cury R. (2017). The Influence of Femoral Tunnel Length on Graft Rupture after Anterior Cruciate Ligament Reconstruction. J. Orthop. Traumatol..

[25] Bullón A., Bravo E., Zarbahsh S., Barco R. (2007). Reconstruction after chronic extensor pollicis longus ruptures: A new technique. Clin. Orthop. Relat. Res..

[26] Gulati H.S., Ray B., Sushma, D’Souza A.S., Kumar N. (2014). Morphometry of the Deep Muscles of the Extensor Compartment of the Forearm and Related Variations. Eur. J. Anat..

[27] Duque-Colorado J., García-Orozco L., Riveros A., Del Sol M. (2025). Scapular notch, spinoglenoid notch, and scapular dimensions: Implications on the safe zone of the suprascapular nerve. Anat. Cell Biol..

[28] Duque-Colorado J., Alzate-Mejia O.A., del Sol M. (2025). Morphometry of the Scapular Notch and Its Clinical Implication in Suprascapular Nerve Entrapment. Diagnostics.

[29] Iwanaga J., Singh V., Ohtsuka A., Hwang Y., Kim H.J., Moryś J., Ravi K.S., Ribatti D., Trainor P.A., Sañudo J.R. (2021). Acknowledging the use of human cadaveric tissues in research papers: Recommendations from anatomical journal editors. Clin. Anat..

[30] Iwanaga J., Kim H.J., Akita K., Logan B.M., Hutchings R.T., Ottone N., Nonaka Y., Anand M., Burns D., Singh V. (2025). Ethical Use of Cadaveric Images in Anatomical Textbooks, Atlases, and Journals: A Consensus Response From Authors and Editors. Clin. Anat..

